# Changes in photosynthesis and grazing facilitate growth of a mixotrophic protist under ocean acidification and warming

**DOI:** 10.1111/nph.71315

**Published:** 2026-07-02

**Authors:** Shai Slomka, Jolanda M. H. Verspagen, Rocio B. Rodriguez‐Perez, Jef Huisman, Susanne Wilken

**Affiliations:** ^1^ Department of Freshwater and Marine Ecology (FAME), Institute for Biodiversity and Ecosystem Dynamics (IBED) University of Amsterdam PO Box 94920 Amsterdam 1090 GE the Netherlands

**Keywords:** chrysophytes, mixotrophy, ocean acidification, ocean warming, *Ochromonas* CCMP2951, phytoplankton

## Abstract

- Mixotrophic protists capable of both photosynthesis and phagotrophy are key members of marine plankton communities. Yet, little is known about their responses to the combination of ocean acidification and warming.
- A marine mixotrophic chrysophyte, *Ochromonas* CCMP2951, was subjected to two levels of pCO_2_ (300 and 800 ppm, resulting in pH of 8.2 and 7.8) and temperature (21°C and 26°C) in a factorial design.
- Enhanced growth rates were observed in both the high CO_2_ and high temperature treatments, while cell size significantly decreased with temperature. Strongly decreased cellular phosphorus content led to increased N : P and C : P ratios of *Ochromonas* with temperature. Furthermore, warming increased grazing rates, while elevated CO_2_ reduced the Chl content but increased photosynthetic carbon acquisition, albeit only at low temperature. The combination of warming and elevated CO_2_ had antagonistic effects on the balance between autotrophic and heterotrophic carbon acquisition, keeping the net role of this mixotroph in the marine carbon cycle stable.
- Altogether, both the direct stimulation of growth and the indirect effects of altered stoichiometry may favor mixotrophs under future ocean conditions. However, their contribution to future carbon cycling in complex natural communities will need further study.

## Graphical abstract

Grazing rates of *Ochromonas* CCMP2951 expressed as carbon obtained per mean cell volume (a) and C_aut/het_‐photosynthetically fixed carbon divided by carbon obtained through grazing (b) in the four experimental treatments.

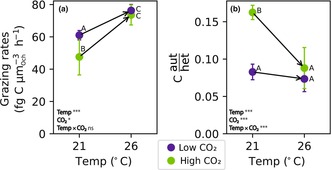

## Introduction

Increasing concentrations of greenhouse gases, specifically, a rise in CO_2_ concentrations from 280 to 420 ppm due to anthropogenic activities, have contributed to an increase in the global temperature of almost 1.3°C above the 20^th^ century average (NOAA National Centers for Environmental Information, 2025). The oceans serve both as a heat and carbon sink. Heat absorption causes ocean warming, while the enhanced CO_2_ influx into the ocean causes ocean acidification, where the decrease in ocean pH is accompanied by increasing concentrations of total dissolved inorganic carbon (DIC), and a shift in DIC‐speciation toward CO_2_ (Doney *et al*., 2009; Findlay *et al*., 2025). Projected increases in global average temperatures range from 1.4°C to 4.4°C by 2100, depending on the Intergovernmental Panel on Climate Change (IPCC) scenario (Calvin *et al*., 2023). Yet, recent marine heatwaves have already resulted in sea surface temperature anomalies several degrees above the long‐term average (Oliver *et al*., 2021; Capotondi *et al*., 2024; England *et al*., 2025). Similarly, increasing pCO_2_ and declines in pH are already detected globally (Ma *et al*., 2023; Findlay *et al*., 2025), while the magnitude of these trends varies regionally and also the seasonal variability in pH and carbonate chemistry is predicted to increase (Kwiatkowski & Orr, 2018). Such drastic changes to the ocean's physical and chemical properties are likely to result in severe changes to marine ecosystems, including shifts in species diversity, species distribution and ecosystem functions (Hoegh‐Guldberg & Bruno, 2010). In turn, changes in the structure and productivity of marine ecosystems can have strong implications for the global carbon cycle, due to the major role of marine microorganisms in carbon transfer (Cavicchioli *et al*., 2019). In particular, phytoplankton primary productivity lies at the basis of the biological carbon pump, which is a main driver of the carbon sequestration capacity of the oceans (Nowicki *et al*., 2022). A change in primary productivity will thus affect the carbon sequestration capacity of marine ecosystems, with potential feedbacks on climate change.

Over the past decades, it has been recognized that a large proportion of the eukaryotic primary producers in the plankton are not strictly autotrophic. Many of them also feed on other microbes via phagocytosis to heterotrophically sustain their growth and metabolism. The importance of these phagotrophic mixotrophs, also referred to as mixoplankton (Flynn *et al*., 2019; Glibert & Mitra, 2022), has been gaining attention (Caron, 2016; Flynn *et al*., 2019), as they have been shown to play a central role in many marine ecosystems. For instance, mixotrophs dominate bacterivory, while also contributing significantly to primary productivity in the oligotrophic gyres (Zubkov & Tarran, 2008; Jardillier *et al*., 2010; Hartmann *et al*., 2012), which are predicted to expand due to climate change (Polovina *et al*., 2008). In coastal ecosystems, mixotrophs can also be abundant (Edwards, 2019), constitute key bacterivores (Unrein *et al*., 2007, 2014) and sometimes contribute to the formation of algal blooms (Harrison *et al*., 2011; Flynn *et al*., 2018). Theoretical work further suggests that ocean acidification will facilitate larger mixotrophic blooms that would enhance primary production (Flynn & Mitra, 2023). As mixotrophic metabolism relies on both autotrophic CO_2_‐consuming processes and heterotrophic CO_2_‐releasing processes, potential shifts in their trophic balance due to climate change may cause an alteration of their role in the carbon cycle.

Temperature appears to be an important regulator of the balance between heterotrophy and autotrophy in mixotrophs. Mixotrophs were previously shown to increase their grazing rates and become more heterotrophic with increasing temperatures (Wilken *et al*., 2013; Liu *et al*., 2021; Lepori‐Bui *et al*., 2022; Ahn & Glibert, 2024). This shift could be explained by the stronger temperature dependency of respiration compared with both photosynthesis (Allen *et al*., 2005) and the uptake of inorganic nutrients (Leles & Levine, 2023), consistent with stronger temperature responses of heterotrophs compared with autotrophs (Rose & Caron, 2007). A mixotroph can therefore enhance its temperature response by shifting its resource allocation away from photosynthesis and toward cellular structures required for feeding and digestion, as also predicted theoretically (Gonzalez *et al*., 2022). However, different temperature responses have also been observed. The primarily autotrophic freshwater mixotroph *Dinobryon* did not shift to heterotrophy with warming (Princiotta *et al*., 2016), while a mixotrophic ciliate and dinoflagellate even showed the opposite response and shifted to autotrophy when exposed to a short‐term 6°C warming (Ferreira *et al*., 2022). Both interspecific differences in mixotrophic functional strategies as well as the time frames of warming vs acclimation might thus impact resource allocation strategies and thermal responses.

Ocean acidification has been shown to generally enhance photosynthesis across different phytoplankton taxa (Mackey *et al*., 2015), likely due to the increase in CO_2_ availability. Accordingly, it was previously hypothesized that elevated CO_2_ might facilitate a transition toward more autotrophy in mixotrophs (Caron & Hutchins, 2013). In a recent study, we explored this hypothesis using three mixotrophic strains, and found variable responses, with two strains showing increased growth under ocean acidification. For one of these strains, the increased growth rate was clearly linked to enhanced photosynthetic rates (Slomka *et al*., 2025). Hence, the impacts of ocean acidification and warming on mixotrophs can differ, yet it remains unclear how mixotrophs will respond to the combination of both ocean acidification and global warming anticipated for the future ocean.

Here, we investigate the individual and combined effects of ocean acidification and warming on the mixotrophic chrysophyte *Ochromonas* sp., strain CCMP2951. This coastal isolate was previously shown to be a facultative mixotroph, growing purely heterotrophically in the dark but also capable of photosynthesis to survive extended prey limitation (Moeller *et al*., 2019; Wilken *et al*., 2020). However, it does have an obligate requirement for prey to achieve positive growth rates, and hence cannot grow in a purely phototrophic mode (Wilken *et al*., 2020). Chrysophytes appear to lack the ability to utilize bicarbonate as a carbon source for photosynthesis (Maberly *et al*., 2009) and most likely this strain therefore relies on CO_2_ as the sole source of inorganic carbon. In addition, in a temperature adaptation study, this strain was shown to have increased growth rates at higher temperatures (Lepori‐Bui *et al*., 2022), altogether indicating that it might benefit from future ocean conditions. This highly plastic strain, which can shift its nutritional balance substantially in response to environmental conditions, offers a good opportunity to test the individual and combined effects of warming and ocean acidification on mixotrophic nutrition and physiology. After acclimatizing this strain to two levels of pH/CO_2_ concentrations and temperatures in a factorial design, we measured a multitude of physiological responses. The results show that ocean acidification and warming increase the cellular N : P and C : P ratios of this marine mixotroph, while they affect its balance between auto‐ and heterotrophic carbon acquisition in opposite directions.

## Materials and Methods

### Algal growth and maintenance

The *Ochromonas* coastal isolate CCMP2951 was obtained from the National Center for Marine Algae and Microbiota (NCMA, East Boothbay, ME, USA) and maintained on K medium (Keller *et al*., 1987) without silicate and Tris‐base. This medium was prepared with a seawater base containing 40% natural seawater collected from the North Atlantic (35.80°N–28°W.41, 20 m depth) and 60% artificial seawater. The K medium has been developed specifically for oceanic microalgae and contains both ammonium (50 μM) and nitrate (882 μM) as N‐source and Na_2_‐glycerophosphate (10 μM) as P‐source. This medium was chosen to prevent impaired photosynthetic growth due to potential inability to access standard nutrient sources, such as nitrate, as reported for some strains of *Ochromonas* (Wilken *et al*., 2014; Lie *et al*., 2017). Macronutrient concentrations in this medium are high to avoid nutrient limitation in the experiment. The culture was kept under a 14 h : 10 h, light : dark cycle with a light intensity of 100 μmol photons m^−2^ s^−1^. Cultures were made axenic before the beginning of the experiment using a mixture of antibiotics (Wilken *et al*., 2020). Cultures were supplemented with the heat‐killed bacterium *Cobetia marina* (DSM4741) obtained from the German Collection of Microorganisms and Cell Cultures (DSMZ, Braunschweig, Germany) as described before (Slomka *et al*., 2025). For preparation of heat‐killed *C. marina*, the bacterium was grown on an artificial seawater base supplemented with monosodium phosphate (200 μM), ammonium chloride (3.2 mM), glucose (3.5 mM), Tris–HCL buffer pH 7.5 (4 mM) and a trace metal mix based on the protocol for L1 medium (Guillard & Hargraves, 1993). Cells were harvested at a late exponential phase, heat‐killed at 60°C for 2 h, pelleted and resuspended in the algal K medium.

### Experimental setup

A warming and acidification experiment was performed on CCMP2951, applying two temperatures (21°C and 26°C) and two pCO_2_ levels in the mineral medium (300 and 800 ppm) in a factorial design of four treatments: LTLC (low temperature, low CO_2_), LTHC (low temperature, high CO_2_), HTLC (high temperature, low CO_2_) and HTHC (high temperature, high CO_2_). We note that a higher pCO_2_ results in a lower pH. The indicated pCO_2_ levels in the mineral medium were set before the inoculation of *Ochromonas*, but shifted slightly during the experiments because of photosynthesis and respiration by *Ochromonas*. Specifically, the pCO_2_ in the low CO_2_ treatments increased to *c*. 350 ppm (see the *Results section*). This pCO_2_ level reflects the average atmospheric pCO_2_ of the late 20^th^ century, and is still within the range of seasonal variation in dissolved CO_2_ found in many present‐day marine and especially coastal waters (Baumann & Smith, 2018; Tsao *et al*., 2023; Ono *et al*., 2024), while the high CO_2_ treatment reflects projections for the end of the century in between the high (RCP6.0) and very high emission (RCP8.5) scenarios (Calvin *et al*., 2023). The 5°C difference between temperature treatments exceeds the projected warming of average sea surface temperatures, yet reflects temperature variation encountered over shorter time scales across seasons and during extreme marine heatwaves (Oliver *et al*., 2021). Both temperature treatments are below the temperature optimum of this strain at *c*. 30°C (see Supporting Information Fig. S1 and Methods S1 for experimental details) and thus represent a temperature range over which metabolic rates increase. Each treatment consisted of five experimental replicates.

The experiment was initiated by inoculating CCMP2951 cultures into media pre‐aerated to the treatment pCO_2_ at the treatment temperature in square, preautoclaved polycarbonate bottles to an initial density of 2 × 10^4^ cells ml^−1^. Cultures were kept in two environmental chambers (SANYO, Osaka, Japan), one set to 21°C for the low temperature treatments, and one to 26°C for the high temperature treatments. The temperatures were verified and corrected with an additional thermometer. Light was supplied from fluorescent lamps at 100 μmol photons m^−2^ s^−1^, programmed to a 14 h : 10 h, light : dark cycle. Experimental bottles were always full to the brim to avoid gas exchange and were maintained semi‐continuously by transfers into fresh, preaerated medium at the treatment temperature. Cultures were sampled for bacterial prey and algal cell counts (1 ml samples) every day, at 2 h into the light period of the diel cycle. Based on these daily cell counts (to be described later), heat‐killed bacteria were supplemented each day to a saturating abundance of 1.5 × 10^7^ cells ml^−1^ to avoid prey depletion in the cultures. We used heat‐killed bacteria to prevent changes in carbonate chemistry of the cultures by prey respiration. As *Ochromonas* achieved growth rates in line with previous studies using live prey (Wilken *et al*., 2020), we consider heat‐killed bacteria to be satisfactory food. Cultures were transferred once *Ochromonas* abundances exceeded 4 × 10^4^ cells ml^−1^ (which occurred over a period ranging from 1 to 3 d) and diluted back to 2 × 10^4^ cells ml^−1^ to avoid significant DIC drawdown during the growth period. pH and DIC samples were taken during every transfer from each of the experimental replicates. In addition to the daily cell counts, flow cytometry samples of the cultures before and after each transfer were fixed and flash frozen for later analysis (to be described later). The cultures were maintained in this manner for 18 d, resulting in a total of 9 or 10 transfers in the low or high temperature treatments, respectively. The long duration ensured full acclimatization of cultures before physiological characterization toward the end of the experiment. Cultures were sampled for elemental content: particulate organic phosphorus (POP), carbon (POC) and nitrogen (PON) on the last transfer (performed on the same day for all treatments). Physiological assays (grazing, carbon fixation and Pulse‐Amplitude Modulation (PAM) fluorometry) as well as sampling for pigment content, and microscopy were all performed 3 h into the light period of the diel cycle at the last day of the experiment.

### Media aeration and carbonate chemistry of the experiment

Before inoculation of *Ochromonas*, sterile media for the experiment were bubbled with air containing different pCO_2_ concentrations as described previously (Slomka *et al*., 2025) to reach a pCO_2_ in the medium of 300 ppm in the low CO_2_ treatment and 800 ppm in the high CO_2_ treatment. During aeration, bottles were kept either at room temperature (21°C) – for the low temperature treatment, or in a 26°C water bath – for the high temperature treatment. After inoculation of *Ochromonas*, the carbonate chemistry was monitored by taking pH samples from grown cultures during every transfer and measuring pH with a SenTix® H electrode (Xylem Analytics GmbH, Weilheim, Germany) calibrated with DIN/NIST buffers. DIC samples were filtered through a 0.2 μm syringe filter and stored in gas tight tubes at 4°C until analysis. All DIC samples were measured at the end of the experiment with a vario TOC cube (Elementar Analysensysteme GmbH, Langenselbold, Germany). Alkalinity and pCO_2_ were calculated from DIC and pH using the PCO2SYS python toolbox v.1.8.2 (Humphreys *et al*., 2023), with the temperature of the treatment given as additional input.

### Flow cytometry sample collection and cell enumeration

Sampling for flow‐cytometric cell enumeration always included fixation with a mixture of glutaraldehyde (0.25% final concentration) and Pluronic F68 (0.01% final concentration) followed by 20 min dark incubation (Marie *et al*., 2014). Samples for routine daily counts were analyzed immediately following this procedure and used to decide on transfers of cultures during the experiment. Additionally, samples taken before and after each transfer were flash frozen in liquid nitrogen and stored in −80°C until analysis. This procedure provides the most accurate cell counts and was used to calculate growth rates presented in the manuscript. *Ochromonas*, heat‐killed bacteria and fluorescently labeled bacteria (FLB) were enumerated using a CytoFLEX flow cytometer (Beckman Coulter, Brea, CA, USA). For enumeration of *Ochromonas*, samples were run undiluted, and a trigger of red fluorescence (690/50 nm) was used. *Ochromonas* were gated based on red‐fluorescence and forward scatter properties. FluoresbriteTM Polychromatic Red 2.0 μm Microspheres (Polysciences, Warrnington, PA, USA) were added to all stored samples as internal standards. This allowed normalization of the forward scatter of the *Ochromonas* population in each sample to the beads' forward scatter and facilitated comparison between samples. For bacterial enumeration, samples were diluted with a Tris‐EDTA buffer and stained with SYBR Green I at a 5 × 10^3^ dilution of the commercial stock (Molecular Probes‐Invitrogen, Carlsbad, CA, USA) followed by 15 min incubation in darkness. Samples were measured with a trigger of green fluorescence (525/40 nm) and gated based on green fluorescence and forward scatter properties.

### Growth rate calculation

Growth rates were calculated based on flow‐cytometric enumeration of *Ochromonas* cells before and after transfer. For each replicate, the specific growth rate was calculated for each of the last three transfers of the experiment in the following manner:

(Eqn 1) $$\mathrm{SGR}=\frac{\log_{e}\left(x_{t+\Delta t}\right)-\log_{e}\left(x_{t}\right)}{\Delta t}$$

where $x_{t}$ corresponds to the cell count before the growth period, $x_{t+\Delta t}$ corresponds to the cell count after the growth period and Δt is the time between transfers in days. Then, for each biological replicate, the specific growth rates of the last three transfers were averaged to obtain a single mean specific growth rate.

### Mean cell volume calculation

Cell volume was calculated from 2‐D measurements of microscopy cell images. Microscopy samples were collected on the last day of the experiment and preserved with 1% alkaline Lugol's solution. Samples were stored at 4°C until analysis. For analysis, samples were pipetted into a 1‐ml sedimentation chamber and were allowed to sediment in the dark for at least 3 h, which was confirmed to allow for full settling of the *Ochromonas* cells. Sedimentation chambers were examined under an inverted microscope (Leica DM IRB, Wetzlar, Germany), and 30 cell images were taken for each biological replicate, using a 630× magnification. Pictures were then analyzed with ImageJ and two dimensions were measured – the height and diameter of the cells. The cell volume was then calculated using a geometric formula for a spheroid cell shape (Hillebrand *et al*., 1999):

(Eqn 2) $$V=\frac{1}{6}\pi d^{2}h$$

where d is the diameter and h is the height measured. The mean cell volume per biological replicate was calculated based on the cell volume of 30 cells.

### Cellular elemental content

Samples for analysis of cellular elemental content were first prefiltered over a 10‐μm polycarbonate filter (47 mm, Whatman Cyclopore™; Cytiva, Wilmington, DE, USA), before filtration over the designated filters, either precombusted GF/D filters for POC/PON analysis (25 mm, Whatman), or acid rinsed 3‐μm polycarbonate filters for POP analysis (25 mm, Whatman Cyclopore™). The prefiltration step was added to improve the accuracy of cell counts by breaking down potential large cell aggregates and clearing large bacterial prey aggregates that cannot be enumerated well and might bias the analyses. Samples for flow‐cytometric cell enumeration were taken before and after prefiltration for each replicate. In addition, to account for the presence of the bacterial prey on the filters, and potential loss of *Ochromonas* cells, samples before and after filtration were fixed and stored for flow‐cytometric enumeration of bacteria and algal cells. Bacterial prey samples were also filtered (on either precombusted GF/F or acid rinsed 0.2‐μm polycarbonate filters) to calculate bacterial elemental content for correction. All filters were flash frozen and stored at −80°C until analysis. Analysis of POC and PON was performed on the same filter using a solid elemental analyzer (varioEL Cube; Elementar Analysensysteme, Langenselbold, Germany). Before analysis, the filters were freeze‐dried and acidified with 4 M HCL. A calibration curve of sulfanilic acid, ranging from 50 to 500 μg, on precombusted GF/D filters was included in triplicates and was used to determine carbon and nitrogen amounts on the experimental filters. For the POP analysis, filters were freeze‐dried and then transferred to acid rinsed Kimax glass tubes. Ten milliliters of MQ‐water were added, together with 0.2 ml of 5.5 M sulfuric acid and 1 ml of freshly made 8% ammonium persulfate. Tubes were autoclaved at 121°C for 30 min and phosphate concentrations analyzed the following day using an auto‐analyzer (San++ system; Skalar, Breda, the Netherlands).

### Photosynthetic carbon fixation assay

Carbon fixation was measured via ^14^C‐uptake in short‐term (1 h) and long‐term (24 h) incubations, to represent short‐term gross carbon fixation rates and daily net carbon fixation rates, respectively. Each experimental replicate was divided over three glass scintillation vials containing 20 ml each. Two vials of each experimental replicate were spiked with 100 μl of ^14^C‐labeled bicarbonate to a final radioactivity of 0.5 μCi ml^−1^. One of those vials was covered in aluminum foil and served as the dark control. The vials were placed in plastic transparent water baths set to 21°C and 26°C, respectively, and positioned on top of a LED light source of 100 μmol photons m^−2^ s^−1^ programmed to a 14 h : 10 h, light : dark cycle. After 1 h of incubation, two samples were taken from each vial. A 100‐μl sample was added immediately to 4 ml Ultima Gold scintillation cocktail (Revvity, Groningen, the Netherlands) for measurement of total activity (DPM; disintegrations per minute) in a scintillation counter (Hidex 600 SL; Turku, Finland; ${\mathrm{DPM}}_{\text{total}}$ for 1 h samples). A 2‐ml sample was transferred to a new glass vial and subsequently 2 ml of 10% glacial acetic acid in 70% methanol was added. These vials were then dried overnight at 60°C until all liquid had evaporated. This procedure thus retained all nonvolatile organic carbon, including the dissolved fraction. Samples were re‐dissolved in 2 ml of MQ‐water and 10 ml of scintillation cocktail were added for counting in the scintillation counter (${\mathrm{DPM}}_{\text{fixed}}$ = DPM fixed in 1 h samples). After 24 h of incubation, the same process was repeated (${\mathrm{DPM}}_{\text{total}}$ = DPM total 24 h and ${\mathrm{DPM}}_{\text{fixed}}$ = DPM fixed 24 h samples). DPM values obtained were converted into carbon fixation rates over 1 h (representing gross rates) and 24 h (representing net rates) as follows:

(Eqn 3) $$C_{\mathrm{PS}}=\frac{\left(\frac{{\mathrm{DPM}}_{\text{fixed}}-{\mathrm{DPM}}_{\text{fixed dark}}}{{\mathrm{DPM}}_{\text{total}}}\right)\times \left(\frac{\text{Total}\;\mathrm{DIC}}{\Delta t}\right)\times 1.05}{\text{Ochromonas abundance}}$$

where 1.05 is the isotopic discrimination factor, Δt was either 1 h or 24 h for gross and net rates respectively, and TotalDIC was the total dissolved inorganic carbon measured for each sample before the carbon fixation assay. ${\mathrm{DPM}}_{\text{fixed dark}}$ is the value obtained from the dark control. *Ochromonas* abundance measured before the incubation period was used for calculation of cellular gross rates. To calculate cellular net carbon fixation rates, the mean specific growth rate of each replicate was used to predict cell counts after 24 h, and then, an average abundance $\bar{N}$ (Heinbokel, 1978) over the 24 h was used:

(Eqn 4) $$\bar{N}=\frac{N_{t}-N_{0}}{\log_{e}(N_{t)}-\log_{e}\left(N_{0}\right)}$$

where $N_{t}$ is the *Ochromonas* abundance estimated after 24 h and $N_{0}$ is the measured *Ochromonas* abundance at the beginning of the incubation. For comparison between net and gross rates, hourly gross rates were first multiplied by 14 h of light in order to obtain daily gross rates, and subsequently, the daily net rates were expressed as percentage of the daily gross rates.

### Pulse‐amplitude modulation fluorometry

Because of the relatively low cell abundances, cultures had to be concentrated for PAM‐measurements. For this purpose, they were gently filtered over a 25‐mm GF/D filter in a Whatman filtration unit. Filters were left slightly wet and then covered with a cork lid and dark acclimated for 20 min. After dark acclimation, the rest of the water was filtered and a mini‐PAM II sensor attached to a cork lid was placed just above the filters. A light curve was performed on each filter, which included exposure to actinic light for 25 s under the following intensities: 0, 36, 80, 144, 216, 300, 423, 570 and 844 μmol photons m^−2^ s^−1^, each followed by a saturation pulse to measure effective quantum yield of Photosystem II (PSII). The relative electron transfer rate (rETR) for each light level was calculated based on the following equation (Beer *et al*., 2021):

(Eqn 5) rETR=Yield×PAR×0.5

where yield is determined as the effective quantum yield of photochemical energy conversion by Photosystem II measured with the PAM, PAR is the photosynthetically active radiation, and the factor 0.5 assumes an equal distribution of the absorbed PAR over Photosystem I and Photosystem II. Photosynthesis–irradiance curves were constructed from the rETR values. Then, a previously described P‐I model (Eq. 6; Eilers & Peeters, 1988) was fit to each of the light curves using a nonlinear least squares fitting with SciPy (Virtanen *et al*., 2020) and the following parameters were extracted from parameters a, b and c: α – the slope of the curve at low light intensities (Eq. 7), rETR_max_ – the maximal rETR reached under saturating intensities (Eq. 8), and *I*_*k*_ – the half‐saturation light intensity (Eq. 9):

(Eqn 6) $$\text{rETR}=\frac{\mathrm{PAR}}{a{\mathrm{PAR}}^{2}+\text{bPAR}+c}$$

(Eqn 7) $$\alpha =\frac{1}{c}$$

(Eqn 8) $$\text{rETRmax}=\frac{1}{b+2\sqrt{\mathrm{ac}}}$$

(Eqn 9) $$I_{k}=\frac{c}{b+2\sqrt{\mathrm{ac}}}$$

### Grazing assay

For the grazing assay, 20 ml aliquots of each biological replicate were transferred into a glass scintillation vial. First, heat‐killed bacteria were added to each vial to reach a similar abundance in all vials of 1.5 × 10^7^ cells ml^−1^. Then, heat‐killed FLB were added to each vial in a final concentration of roughly 1 × 10^6^ cells ml^−1^. A flow cytometry sample was taken immediately after addition of FLB. Vials were left at their respective environmental chambers for 20 min until an additional flow cytometry sample was taken. Preparation of FLB, analysis of flow cytometry samples and calculation of grazing rates from flow cytometry samples at the two time points of the experiment were performed as described previously (Slomka *et al*., 2025).

### Carbon acquisition balance calculation

For estimation of the gross carbon acquisition balance of each replicate, grazing rates were converted to fg C μm*_Ochr_* ^‐3^ d^‐1^ by multiplying the number of bacteria consumed by their cellular carbon content (130 fg C per cell) derived from the POC measurements of the bacterial prey. We then quantified the balance between autotrophic and heterotrophic gross carbon acquisition (C_aut/het_) by calculating the ratio of carbon obtained by gross photosynthesis (C_PS_) to carbon obtained by grazing (C_GR_). That is:

(Eqn 10) $$C_{\mathrm{aut}/\mathrm{het}}=\frac{C_{\mathrm{PS}}}{C_{\mathrm{GR}}}$$

### Pigment content

Filters from the PAM assay were flash frozen immediately after recording the light curves and stored at −80°C to be used for pigment content analysis. Filters were first freeze‐dried, and then, pigments were extracted and analyzed on HPLC as described previously (Slomka *et al*., 2025). The following pigments were measured: Chla, Chl *c*1 + *c*2, violaxanthin, antheraxanthin, zeaxanthin, diadinoxanthin, diatoxanthin, fucoxanthin and β‐carotene. Chl*a* concentrations were calculated using known cell abundances from flow cytometry samples, and all other pigments were expressed relative to Chla content. Both diadinoxanthin and diatoxanthin were below detection levels.

### Statistical analyses

Statistical analyses were performed using the python packages SciPy and Statsmodels (Seabold & Perktold, 2010; Virtanen *et al*., 2020). Three‐way ANOVAs were used to assess differences in carbonate chemistry between treatments, with temperature, CO_2_, and the presence/absence of *Ochromonas* as main factors and all possible interaction terms. If applicable, nonsignificant interactions and main effects (*P* > 0.05) were removed from the ANOVA models in a hierarchical manner. To assess effects of the treatments on physiological parameters of *Ochromonas*, two‐way ANOVAs were performed with temperature and CO_2_ as main factors and their interaction term. Before each analysis, homogeneity of variances was checked using Levene's test, and normality was tested using the Shapiro–Wilk test. The C : P ratios were ln‐transformed to meet homogeneity of variance. The C : N ratios did not meet the homogeneity of variance requirements for ANOVA even after transformation; therefore, a nonparametric aligned rank transform ANOVA was performed using ARTool (Wobbrock *et al*., 2011). All ANOVAs were followed by *post hoc* comparisons of the means using Tukey's HSD test, with statistical significance set at *P* < 0.05.

## Results

### Carbonate chemistry

Consistent with our experimental design, the mineral medium of the two high CO_2_ treatments (LTHC and HTHC) had a significantly higher dissolved CO_2_(aq) concentration (expressed as pCO_2_) and significantly lower pH than the two low CO_2_ treatments (LTLC and HTLC; Tables 1, S1). DIC in the mineral medium did not differ significantly between the treatments. The low CO_2_ treatments had a slightly higher alkalinity than the high CO_2_ treatments, regardless of temperature or *Ochromonas* growth (Tables 1, S1). Over time, the carbonate chemistry changed slightly in the cultures with *Ochromonas* compared with the mineral medium, potentially due to some air exchange during sampling, but mainly due to the growth of *Ochromonas*. This resulted in some divergence between treatments, such as a higher pCO_2_ in the LTHC treatment than in the HTHC treatment (Table 1). The cultures of the low CO_2_ treatments (LC) had a dissolved CO_2_(aq) concentration equivalent to 332–358 ppm and pH of 8.16–8.20, while the high CO_2_ cultures (HC) ranged between 770 and 880 ppm and pH of 7.79–7.87. In line with recommendations for ocean acidification experiments (Riebesell *et al*., 2011), the DIC drawdown over each growth cycle remained below 2.2% on average in all treatments. The temporal dynamics of pCO_2_, DIC, alkalinity and pH across the full experimental duration are shown in Fig. S2.

**Table 1:** Temperature and carbonate chemistry measured in the mineral medium (without *Ochromonas* CCMP2951) and in the cultures (with *Ochromonas*) throughout the experiment (LTLC – low temperature low CO_2_, LTHC – low temperature high CO_2_, HTLC – high temperature low CO_2_, HTHC – high temperature high CO_2_).

Treatment	Temperature (°C)	pCO_2_ (ppm)	pH	DIC (μmol l^−1^)	Alkalinity (μEq l^−1^)
Mineral medium without *Ochromonas*					
LTLC	21	290 ± 47 (a)	8.25 ± 0.05 (a)	1747 ± 116	2051 ± 115 (a)
LTHC	21	789 ± 37 (b)	7.85 ± 0.04 (b)	1772 ± 162	1902 ± 177 (b)
HTLC	26	287 ± 5 (a)	8.25 ± 0.04 (a)	1673 ± 178	2011 ± 224 (a)
HTHC	26	818 ± 85 (b)	7.84 ± 0.08 (b)	1717 ± 189	1864 ± 218 (b)
Cultures with *Ochromonas*					
LTLC	21	358 ± 12 (a)	8.16 ± 0.01 (c)	1717 ± 11	1972 ± 13 (a)
LTHC	21	883 ± 25 (c)	7.79 ± 0.01 (b)	1733 ± 9	1846 ± 13 (b)
HTLC	26	332 ± 8 (a)	8.20 ± 0.01 (ac)	1657 ± 15	1959 ± 21 (a)
HTHC	26	769 ± 47 (b)	7.87 ± 0.03 (b)	1716 ± 4	1874 ± 12 (b)

For the mineral medium, the mean ± SD is shown for each treatment (*n* = 3 for low CO_2_ treatments and *n* = 4 for high CO_2_ treatments). For cultures, values were first averaged over time within each replicate. The mean ± SD of these biological replicates (*n* = 5) is shown for each treatment. Three‐way ANOVA revealed significant differences in pCO_2_, pH and alkalinity between different treatments (Supporting Information Table S1). Results of the *post hoc* comparisons of the means using Tukey's HSD test are presented in letters within brackets. DIC corresponds to dissolved inorganic carbon.

### Growth rate, size and elemental composition are affected by both warming and ocean acidification

**Fig. 1:**
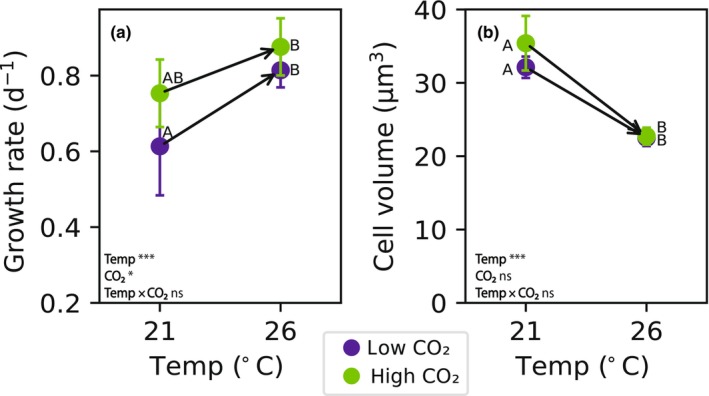
Mean specific growth rate (a) and cell volume (b) of *Ochromonas* CCMP2951 in the four experimental treatments. Arrows extend between the low and the high temperature of each CO_2_. Error bars correspond to the SD from the mean. The statistical significance of the two main effects (Temp and CO_2_) and their interaction (Temp × CO_2_) tested with two‐way ANOVA are indicated (ns, *P* > 0.05; *, *P* < 0.05; ***, *P* < 0.001). Letters represent statistically significant differences between treatments based on Tukey's HSD pairwise *post hoc* comparisons.

Both high temperature and high CO_2_ significantly enhanced the specific growth rate of *Ochromonas* (Fig. 1a; Table S2). Mean specific growth rates ranged from 0.61 ± 0.13 d^−1^ in the LTLC treatment to 0.88 ± 0.08 d^−1^ in the HTHC treatment. The full time course of *Ochromonas* and bacterial abundances over the duration of the experiment is shown in Fig. S3. The cell volume of *Ochromonas* decreased by 32% with warming (Fig. 1b; Table S2), ranging from 34 ± 3 μm^3^ in the low temperature to 23 ± 1 μm^3^ in the high temperature treatments. This strong effect of temperature on cell size was further supported by the normalized forward scatter of the cultures, a proxy for cell size measured by flow cytometry, which showed a similar trend (Fig. S4; Table S2). All physiological indicators discussed in the sections below were therefore normalized to cell volume.

**Fig. 2:**
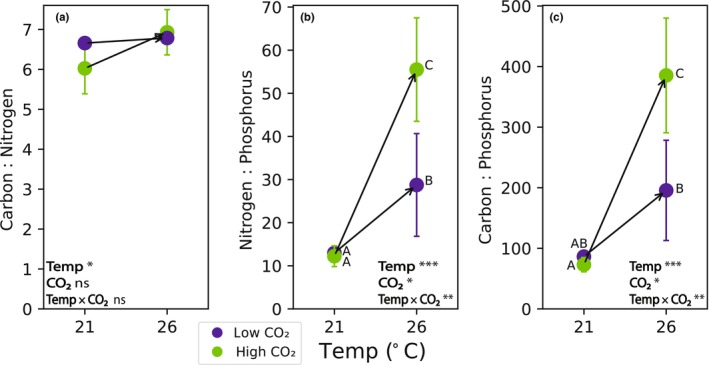
Elemental stoichiometry of *Ochromonas* CCMP2951. Molar ratios of Carbon : Nitrogen (a), Nitrogen : Phosphorus (b) and Carbon : Phosphorus (c) in the different experimental treatments. Arrows extend between the low and the high temperature of each CO_2_. Error bars correspond to the SD from the mean. The statistical significance of the two main effects (Temp and CO_2_) and their interaction (Temp × CO_2_) tested with aligned rank transform ANOVA (for C : N ratios) and two‐way ANOVA (for N : P and C : P ratios) are indicated (ns, *P* > 0.05; *, *P* < 0.05; **, *P* < 0.01; ***, *P* < 0.001). Letters represent statistically significant differences between treatments based on Tukey's HSD pairwise *post hoc* comparisons.

In line with the smaller cell size, the cellular contents of carbon and nitrogen decreased with increasing temperature, while the cellular phosphorus content decreased even more strongly, by 80 to 93% (Fig. S5; Table S2). By contrast, the cellular content of nitrogen and phosphorus increased with higher CO_2_, albeit only in the low temperature treatment (Fig. S5B,C; Table S2). Accordingly, the elemental stoichiometry of the cells also varied with experimental treatments. Specifically, warming caused a major increase in cellular N : P and C : P ratios, while ocean acidification increased the N : P and C : P ratios further at high temperature (Fig. 2; Table S2). Warming was also found to cause a slight increase in C : N ratios, though *post hoc* analysis revealed no significant differences between treatments.

### Changes in photo‐physiology with warming and ocean acidification

**Fig. 3:**
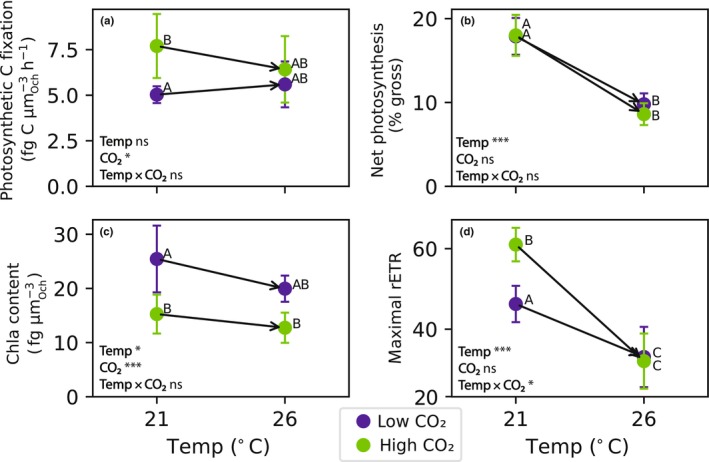
Photo‐physiology of *Ochromonas* CCMP2951: Photosynthetic gross carbon fixation rates normalized to mean cell volume (a), net photosynthetic carbon (C) fixation rates expressed as percent of the gross carbon fixation rates (b), Chl*a* content normalized to cell volume (c), and the maximal relative electron transport rate (rETR) fitted from rapid light curves (d). Arrows extend between the low and the high temperature of each CO_2_. Error bars correspond to the SD from the mean. The statistical significance of the two main effects (Temp and CO_2_) and their interaction (Temp × CO_2_) tested with two‐way ANOVA are indicated (ns, *P* > 0.05; *, *P* < 0.05; ***, *P* < 0.001). Letters represent statistically significant differences between treatments based on Tukey's HSD pairwise *post hoc* comparisons.

Ocean acidification resulted in a significant increase in gross photosynthetic carbon fixation rate, but only at low temperature (Fig. 3a; Table S2), from 5.0 ± 0.5 fg C μm^−3^ h^−1^ to 7.7 ± 1.8 fg C μm^−3^ h^−1^. Net C fixation expressed as percentage of the gross C fixation rate was not affected by ocean acidification but showed a steep decrease with temperature (Fig. 3b; Table S2). Mean net C fixation ranged from 17.9 ± 2.2% of gross C fixation at the low temperature to 9.2 ± 1.4% at the high temperature.

The pigment content and composition of *Ochromonas* also varied with experimental treatments. Chl*a* content decreased with both temperature and ocean acidification (Fig. 3c; Table S2). High temperature significantly increased the ratio of accessory pigments Chl*c* (sum of Chl *c*_1_ and *c*_2_) and fucoxanthin relative to Chla, while these accessory pigments were not affected by the ocean acidification treatment (Fig. S6; Table S2). No significant changes were observed for the photo‐protective pigments of the violaxanthin cycle and *β*‐carotene (Fig. S6; Table S2).

Photosynthesis–irradiance curves (Fig. S7A) revealed a significantly lower maximal rETR at saturating irradiance (Fig. 3d; Table S2) and lower initial slope (α) of the light curve (Fig. S7B; Table S2) in the high temperature treatment, implying a decreased photosynthetic efficiency of Photosystem II with warming. Conversely, maximal rETR was significantly higher in the ocean acidification treatment, but only at 21°C, indicative of enhanced photosynthetic electron transport at elevated pCO_2_ (Fig. 3d; Table S2). No treatment effect was found on the half‐saturation light intensity *I*_*k*_ (Fig. S7C; Table S2).

### Opposite effects of ocean acidification and warming on the carbon acquisition balance

**Fig. 4:**
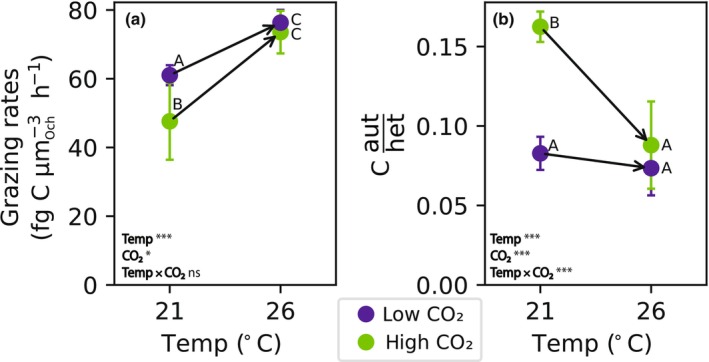
Grazing rates of *Ochromonas* CCMP2951 expressed as carbon obtained per mean cell volume (a) and $C_{\mathrm{aut}/\mathrm{het}}$‐ photosynthetically fixed carbon divided by carbon obtained through grazing (b) in the four experimental treatments. Arrows extend between the low and the high temperature of each CO_2_. Error bars correspond to the SD from the mean. The statistical significance of the two main effects (Temp and CO_2_) and their interaction (Temp × CO_2_) tested with two‐way ANOVA are indicated (ns, *P* > 0.05; *, *P* < 0.05; ***, *P* < 0.001). Letters represent statistically significant differences between treatments based on Tukey's HSD pairwise *post hoc* comparisons.

Grazing rates increased with temperature (Fig. 4a; Table S2) and, conversely, decreased with ocean acidification, but only at the low temperature. The balance between autotrophic and heterotrophic carbon acquisition (C_aut/het_) showed that *Ochromonas* acquired *c*. 10 times more carbon through grazing than through gross photosynthetic carbon fixation (Fig. 4b). This ratio increased with ocean acidification but only at low temperature (Fig. 4b; Table S2). Conversely, it decreased with temperature but only in the ocean acidification treatment. Due to these interactive effects, *Ochromonas* retained a similar carbon acquisition balance in the combined treatment with ocean acidification and high temperature as in the control treatment.

## Discussion

Our results show a positive growth response of *Ochromonas* CCMP2951 to both ocean acidification and to warming, yet these two drivers had contrasting effects on the trophic balance between autotrophic and heterotrophic carbon acquisition. Ocean acidification increased relative autotrophy in the mixotroph by higher carbon fixation rates and lower grazing rates, while warming induced increased heterotrophy due to higher grazing rates despite similar carbon fixation rates. In the combined treatment, the effects of ocean acidification were diminished at high temperature, so that *Ochromonas* displayed a similar balance between autotrophy and heterotrophy as in the control (low CO_2_ and low temperature) treatment.

Rising CO_2_ concentrations are expected to primarily affect the dark reaction of photosynthesis, as they increase the substrate availability for carbon fixation (Raven & Johnston, 1991). Yet, rising CO_2_ can also impact the light reaction of photosynthesis, as the increased demand for ATP and reducing equivalents to support increased rates of carbon fixation diminishes the accumulation of excess excitation energy at PSII, thereby enhancing photosynthetic electron transport (Baker, 2008; Zavřel *et al*., 2018) and thus maximal rETR as observed in the ocean acidification treatment at 21°C. The higher relative electron transport rate (rETR) was accompanied by a decreasing Chla content, which might have kept absolute electron transport rates stable.

Our results also show an increased CO_2_ fixation efficiency in the ocean acidification treatment, as less Chl was required for higher rates of carbon fixation (Fig. 3a,c). A reduced Chl content in response to elevated CO_2_ has been observed in some plants (Thron *et al*., 1997; Sicher, 2011) and microalgae (Hennon *et al*., 2017), but is certainly not a universal response (Hennon *et al*., 2017; Lupitu *et al*., 2022). A potential explanation for the increased efficiency of the dark reaction might be lower rates of photorespiration at higher CO_2_ availability. Photorespiration is particularly common in algae without carbon concentrating mechanisms (CCMs), such as chrysophytes (Bhatti & Colman, 2011). Photorespiration consumes ATP and NADPH and also produces toxic phosphoglycolate that needs to be metabolized. Lowered rates of photorespiration at high CO_2_ could therefore increase the efficiency of photosynthesis and diminish energetic costs for metabolizing phosphoglycolate, which would enable higher rates of carbon fixation with less Chl, as observed in this study, and likely also with less investment into RuBisCO. Our findings are in good agreement with the recent model of Moeller *et al*. (2024) investigating optimal metabolic strategies of mixotrophs. Their model predicts a major trade‐off in resource acquisition, where an increased C uptake efficiency would allow mixotrophs to lower investments into light harvesting, which can free up resources to be invested into growth or phagotrophic nutrient acquisition instead.

Warming also affects the dark reaction of photosynthesis, as it increases enzymatic reaction rates up to an optimum temperature (Allen *et al*., 2005). Our results show that temperature did not affect gross carbon fixation rates, while it negatively affected both the Chla concentration and maximal rETR. These changes suggest decreased capacity for light harvesting and for photosynthetic electron transfer at higher temperatures, although lower Chla concentrations might have been partly compensated by the relative increase in accessory pigments Chl *c* and fucoxanthin, which are both involved in light harvesting for photosynthesis. Thus, a potential increase in carbon fixation rates, as expected by increasing enzyme activity with warming (Allen *et al*., 2005), might have been countered by a decreased investment and potential alteration of the photosynthetic machinery. This is in contrast to purely autotrophic phytoplankton, which typically increase their investment into photosynthesis with temperature in order to increase rates of photosynthesis in par with increasing rates of respiration (Leles & Levine, 2023). Yet, mixotrophs have the option to invest in phagotrophy instead, and similar changes in the light and dark reactions of photosynthesis were previously described for a freshwater mixotrophic *Ochromonas* species shifting toward heterotrophy (Wilken *et al*., 2013).

At low temperature, net rates of carbon fixation constituted a relatively small percentage (17.9%) of the gross carbon fixation rates. This percentage is lower than typically reported for pure autotrophs (Halsey *et al*., 2013; Halsey & Jones, 2015), which suggests that access to an organic carbon source resulted in a disproportionately large allocation of photosynthetically fixed carbon to fuel respiration in this mixotrophic organism. However, there are some caveats in this interpretation. First, our estimates of gross carbon fixation are based on short (1 h) incubations, as longer incubations can be biased by rapid respiration of recently fixed carbon (Milligan *et al*., 2015). Yet, this bias cannot be entirely excluded even over 1 h. Hence, our gross fixation values represent minimum estimates, and our finding that net carbon fixation is only 17.9% of gross fixation might still be an overestimate. Second, the short‐term incubations represent only a snapshot in time, and gross carbon fixation rates may vary over the diel cycle. Hence, the 17.9% estimate should be interpreted with caution.

The observed further decrease in net carbon fixation as a percentage of gross carbon fixation at high temperature likely reflects increased respiration rates, a well‐known response to warming across a broad range of organisms (Brown *et al*., 2004), including *Ochromonas* (Lepori‐Bui *et al*., 2022). Increased grazing rates of *Ochromonas* CCMP2951 with warming are in accordance with previous studies showing a similar shift toward heterotrophy in mixotrophic organisms (Wilken *et al*., 2013; Liu *et al*., 2021). The relative decline in net carbon fixation might thus be a direct result of heterotrophic metabolism responding more strongly to temperature than autotrophic processes, in line with the metabolic theory of ecology (Allen *et al*., 2005). This shift to heterotrophic metabolism might be further amplified by altered resource allocation toward phagotrophy, as has also been predicted for evolutionary adaptation of mixotrophs to warming, if sufficient prey is available to support phagotrophy (Gonzalez *et al*., 2022).

The close association between increasing pCO_2_ and the decline in pH often prevents teasing apart the individual effects of pH vs pCO_2_ on physiological responses. Positive effects of ocean acidification on phytoplankton growth and photosynthesis are commonly observed (Mackey *et al*., 2015) and ascribed to CO_2_‐fertilization effects. On the other hand, direct pH effects have been reported to increase grazing rates in a freshwater mixotroph (Xu *et al*., 2023), potentially via increased motility (Kim *et al*., 2013) and thus higher encounter rates with prey, while the opposite effect of decreased motility has also been described (Wang *et al*., 2020). The increasing rates of carbon fixation and growth found in *Ochromonas* CCMP2951 under ocean acidification, together with a slight decline in grazing rates, indicate pCO_2_ as the primary driver of physiological adjustments in this species. The growth of freshwater chrysophytes is indeed known to be very sensitive to pCO_2_, as they lack CCMs, suggesting reliance on CO_2_ as the sole inorganic carbon substrate (Maberly *et al*., 2009; Wolfe & Siver, 2013). Most likely, the lack of CCMs is widespread among chrysophytes and applies to marine strains as well. Yet even in species with highly efficient CCMs, increasing pCO_2_ can relieve the reliance on these energy demanding processes (Raven *et al*., 2011). Nevertheless, we cannot exclude the possibility of pH effects. Because heterotrophy and photosynthesis have opposite impacts on near‐cell pH, it has for instance been suggested that their regulation in mixotrophs might aid in maintaining pH homeostasis (Flynn & Mitra, 2023). The increased rates of photosynthesis in *Ochromonas* could thus also serve to balance near‐cell pH under ocean acidification.

Our results suggest that warming will shift the metabolism of mixotrophs toward relatively more heterotrophy, while ocean acidification will shift it toward more autotrophy. Yet, what the balance of these two opposing effects will be is difficult to predict and might vary between species and ecosystems. Both temperature and CO_2_ responses are nonlinear and impacts thus differ between the higher, optimal or lower end of the CO_2_ and thermal niches. Furthermore, the rate of warming and acidification expected with climate change, as well as their variability over diel and seasonal cycles, differs between ecosystems (Levitus *et al*., 2000; Hofmann *et al*., 2011; Yao *et al*., 2017). Physiological shifts in the trophic balance of mixotrophs due to acclimation as assessed here appear to differ from both short‐term stress responses to, for example rapidly changing temperatures (Ferreira *et al*., 2022; Lepori‐Bui *et al*., 2022) and longer‐term evolutionary changes in response to warming (Lepori‐Bui *et al*., 2022). This is in line with observations for phytoplankton in general, for which evolutionary adaptations to warming and ocean acidification are much better studied (Schlüter *et al*., 2014; Thangaraj & Sun, 2023; Wu *et al*., 2025) and which often involve adjustments of metabolic rates that differ from those seen during physiological acclimation (Padfield *et al*., 2016; Jin *et al*., 2022). In reality, changes of these different processes at these different timescales will be superimposed. Shifts in the future nutrition of mixotrophs can therefore not be inferred solely from physiological experiments and the likelihood of different climate change scenarios, but will also require further insights into the biological variability of species responses and their potential evolutionary adaptations.

Mixotrophs have diverse phylogenies and functional strategies (Li *et al*., 2022). *Ochromonas* CCMP2951 was isolated from a coastal site at the western boundary of the North Pacific subtropical gyre. Our present findings and previous results (Wilken *et al*., 2020) show that this strain obtained much more carbon from grazing than from photosynthesis, and hence is a chrysophyte with a relatively heterotrophic lifestyle. Mixotrophs with high grazing rates have an advantage in strongly stratified surface waters with low nutrient but high light availability, as both found empirically (Edwards *et al*., 2023) and predicted theoretically (Moeller *et al*., 2024) for subtropical ocean gyres. High rates of phagotrophy could relieve both the need to invest into photosynthesis and inorganic nutrient uptake, thus increasing the competitive advantage of mixotrophs in oligotrophic waters. In *Ochromonas* CCMP2951, grazing rates of 10–15 bacteria cell^−1^ h^−1^ observed in our experiment together with measured bacterial C, N and P quotas of 11.15 fmol C per cell, 2.26 fmol N per cell and 0.15 fmol P per cell are sufficient to meet and exceed the cellular quotas of carbon, nitrogen and phosphorus (Fig. S5) if comparable grazing rates are sustained over the course of the day. Because these grazing rates were reached despite high nutrient and light availability in the experiment, we expect the responses to warming and ocean acidification observed in this strain to be more sensitive to variation in prey availability than to variation in nutrient and light availability. Because light, nutrient and prey availability will be impacted by intensified stratification under warming, the interactive effects of these concurrent environmental changes might alter the responses to ocean acidification and warming. Such interactions are further expected to differ between functionally distinct mixotrophs, with relatively more autotrophic lifestyles likely being more responsive to changes in nutrient availability. How functionally diverse mixotrophs will respond to future ocean conditions thus remains an open question.

The marked increase in the N : P and C : P ratios with temperature observed in our experiments is consistent with previous phytoplankton studies (Hessen *et al*., 2005; Martiny *et al*., 2013; Thrane *et al*., 2017; Yvon‐Durocher *et al*., 2017; Tanioka & Matsumoto, 2020) and driven by a strong decrease in cellular P‐content (80–93%), relative to the decreases in cell size (32%) as well as cellular N (60%) and C content (59%). Given that RNA‐derived phosphorus is the largest nonstorage cellular P pool (Raven, 2013), a plausible physiological explanation is based on increasing rates of biosynthesis with temperature, requiring a lower number of P‐rich ribosomes to produce the same amount of protein (Toseland *et al*., 2013). Hence, cells will have lower P requirements at elevated temperature. Yet, this mechanism alone is unlikely to explain the *c*. fourfold decline in phosphorus per biovolume and other mechanisms might also be at play. These could include decreased P‐storage capacity due to the smaller cell size at elevated temperature, dilution of P‐storage by the enhanced growth rate, and perhaps partial substitution of phospholipids with non‐P‐containing glycerol glycolipids or sulfolipids (Van Mooy *et al*., 2009; Huang *et al*., 2023). Our results show that this temperature‐driven change in N : P and C : P stoichiometry is not restricted to autotrophic phytoplankton but even occurs in relatively heterotrophic mixotrophs such as our *Ochromonas* strain, which obtains most of its carbon (and nutrients) from grazing. Moreover, our results also show that ocean acidification results in an even further increase in the N : P and C : P ratio at high temperature. The high stoichiometric flexibility of *Ochromonas* deviates from the common view that the elemental composition of mixotrophs is relatively homeostatic in comparison with autotrophic organisms (Schenone *et al*., 2024). A similar shift in elemental stoichiometry with elevated temperature and CO_2_ was observed in the mixotrophic bloom‐forming raphidophyte *Heterosigma akashiwo* but not in the dinoflagellate *Prorocentrum minimum* (Fu *et al*., 2008), indicating that mixotrophic species may vary in their stoichiometric flexibility.

An important implication of these findings is that, in comparison with homeostatic zooplankton, stoichiometrically flexible mixotrophs are likely to increase their competitive ability in P‐limited environments with warming, because they increase their grazing rates and concomitantly decrease their P requirements. A higher C : P ratio at elevated temperature also implies that these mixotrophs will be of lower food quality for potential predators. This combination of an enhanced competitive ability and lower nutritional value at high temperature may help to explain why mixotrophic protists are often the dominant grazers in oligotrophic subtropical gyres (Hartmann *et al*., 2012) and indicates that they will benefit from expansion of these gyres by ocean warming. While increased C : P ratios of mixotrophs might indicate that more carbon could be exported in P‐depleted waters, the observed decrease in cell size with warming, which is a general trend among protists (Atkinson *et al*., 2003), is likely to have an opposite and perhaps bigger effect on the efficiency of the biological carbon pump, as both carbon export (Guidi *et al*., 2009; Luan *et al*., 2026) and trophic transfer efficiency (Marrec & Menden‐Deuer, 2024) tend to decrease for smaller cells.

In conclusion, our results demonstrate that the role of mixotrophs in the carbon cycle of future oceans is likely to be affected by both ocean acidification and global warming, and that understanding the interaction between these will be crucial for projecting future shifts in the trophic balance of mixotrophs as well as potential impacts cascading through the microbial food webs that control marine carbon cycling.

## Competing interests

None declared.

## Author contributions

SS, SW, JMHV and JH conceptualized the project and discussed all data. RBRP performed thermal niche experiments. SS designed and performed the main experiments. SS analyzed data with contributions from RBRP and JMHV. SS and SW wrote the manuscript with contributions from all authors.

## Disclaimer

The New Phytologist Foundation remains neutral with regard to jurisdictional claims in maps and in any institutional affiliations.

## Supporting information


**Fig. S1** Thermal niche of *Ochromonas* CCMP 2951.
**Fig. S2** Carbon chemistry parameters over time.
**Fig. S3** *Ochromonas* and bacterial abundances over time.
**Fig. S4** Normalized forward scatter of *Ochromonas* in the four experimental treatments.
**Fig. S5** Elemental composition of *Ochromonas* in the four experimental treatments.
**Fig. S6** Relative pigment composition of *Ochromonas* in the four experimental treatments.
**Fig. S7** Rapid light curve results of *Ochromonas* CCMP2951 in the four experimental treatments.
**Methods S1** Supplementary materials and methods.
**Table S1** Results of three‐way ANOVA on carbonate chemistry parameters in the experimental treatments.
**Table S2** Results of two‐way ANOVA on physiological parameters of *Ochromonas*.
Please note: Wiley is not responsible for the content or functionality of any Supporting Information supplied by the authors. Any queries (other than missing material) should be directed to the *New Phytologist* Central Office.

## Data Availability

The data supporting this study are available in figshare: doi: 10.6084/m9.figshare.31341967.
