# Supplementary material for: Changes in photosynthesis and grazing facilitate growth of a mixotrophic protist under ocean acidification and warming
Source: New Phytol. 2026 Jul 2;251(5):2574–87. doi: 10.1111/nph.71315 (PMC13443156; doi:10.1111/nph.71315)
Supplement: Supplementary file 1 [file NPH-251-2574-s001.pdf]

New Phytologist Supporting Information

Article title:

Changes in photosynthesis and grazing facilitate growth of a mixotrophic protist under ocean acidification and warming

Authors:

Shai Slomka, Jolanda M. H. Verspagen, Rocio B. Rodriguez-Perez, Jef Huisman and Susanne Wilken*

Department of Freshwater and Marine Ecology (FAME), Institute for Biodiversity and Ecosystem Dynamics (IBED), University of Amsterdam, P.O. Box 94920, 1090 XH, Amsterdam, The Netherlands

*Corresponding author: Susanne Wilken

Email: s.wilken@uva.nl

Article acceptance date: 5 May 2026

Methods S1: Supplementary Materials and Methods

Two preliminary experiments were performed to characterize the thermal niche of *Ochromonas* CCMP2951. In the first experiment growth rates were quantified in duplicate at seven temperatures ranging from 12.3 to 36.0 °C and in the second growth was quantified in five replicates at five temperatures ranging from 18.7 to 32.5 °C. These temperatures were reached in a temperature gradient incubator, consisting of an aluminum block heated on one side and cooled on the other to establish the temperature gradient. This block contained holes to hold 20mL glass scintillation vials in which cultures were grown and was placed onto an 8-color LED light source illuminating cultures from below with an intensity of 100μmol quanta m^{-2} s^{-1} and a light:dark cycle of 14h:10h. Cultures were grown in L1 medium (Guillard & Hargraves, 1993) and fed with heat-killed bacterial prey prepared from cultures of *Cobetia marina* as explained in the main text. In an acclimatization phase of one week, cultures were inoculated to a density of $2x10^4$ cells mL^{-1} and fed with heat-killed bacteria to an abundance of $2x10^7$ cells mL^{-1} every second day. After this acclimatization phase cultures were diluted back to their original abundance followed by sampling and feeding every day, now adding bacterial prey to an abundance exceeding the measured *Ochromonas* abundance 100 times. Cultures were maintained like this for another week. Samples were taken each morning 1h after lights-on, fixed with glutaraldehyde (final concentration of 0.25%) and dark incubated for 15 minutes prior to counting on an Accuri C6 flow cytometer (BD Biosciences, San Jose, CA, USA). Growth rates were quantified by linear regression of the ln-transformed abundances over time for the period in which exponential growth was observed.

We used an optimum function to describe growth rate (μ) as a function of temperature (T) (Eppley 1971; Norberg 2004):

$$\mu = a\ e^{bT} \left(1 - \left(\frac{T-z}{w/2}\right)^2\right) \qquad \text{Eq. S1}$$

where w is the thermal niche width, a and b are parameters of the classic Eppley curve and z is the temperature at which the growth rate is equal to the classic Eppley curve. We fitted the model to the data using the pipeline described in Padfield et al. 2021, using the model function thomas_2012() of the R package rTPC.

Supplementary Figures and Tables

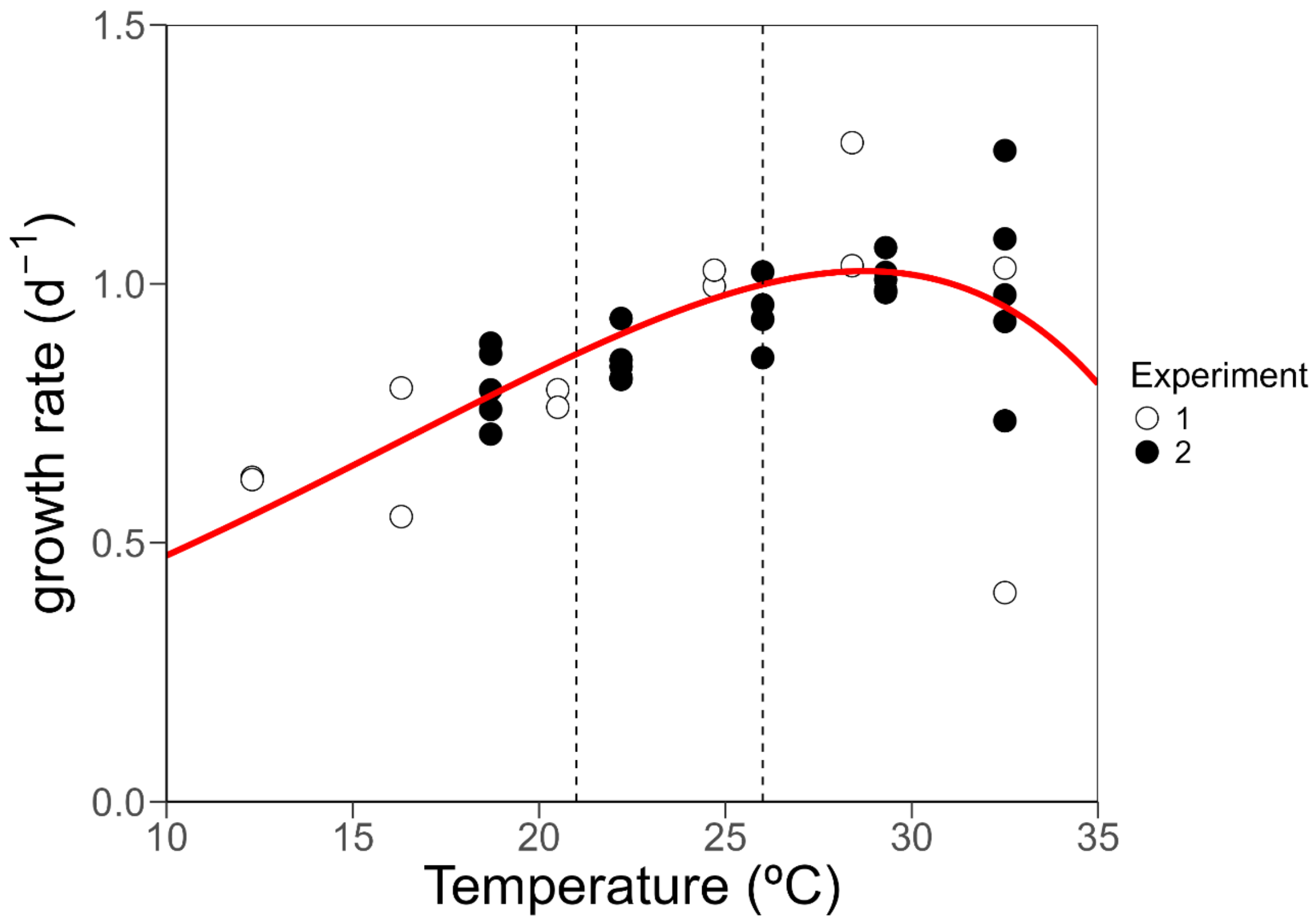

Figure S1: Growth rate of *Ochromonas* CCMP2951 as a function of temperature. Dots show the measured growth rates (n=37). The red line represents the least-squares fit of equation S1 to the data where $a = 0.25527$, $b = 0.06285$, $z = 12.300$ and $w = 56.328$. Vertical dashed lines represent the two temperatures applied in our main experiments.

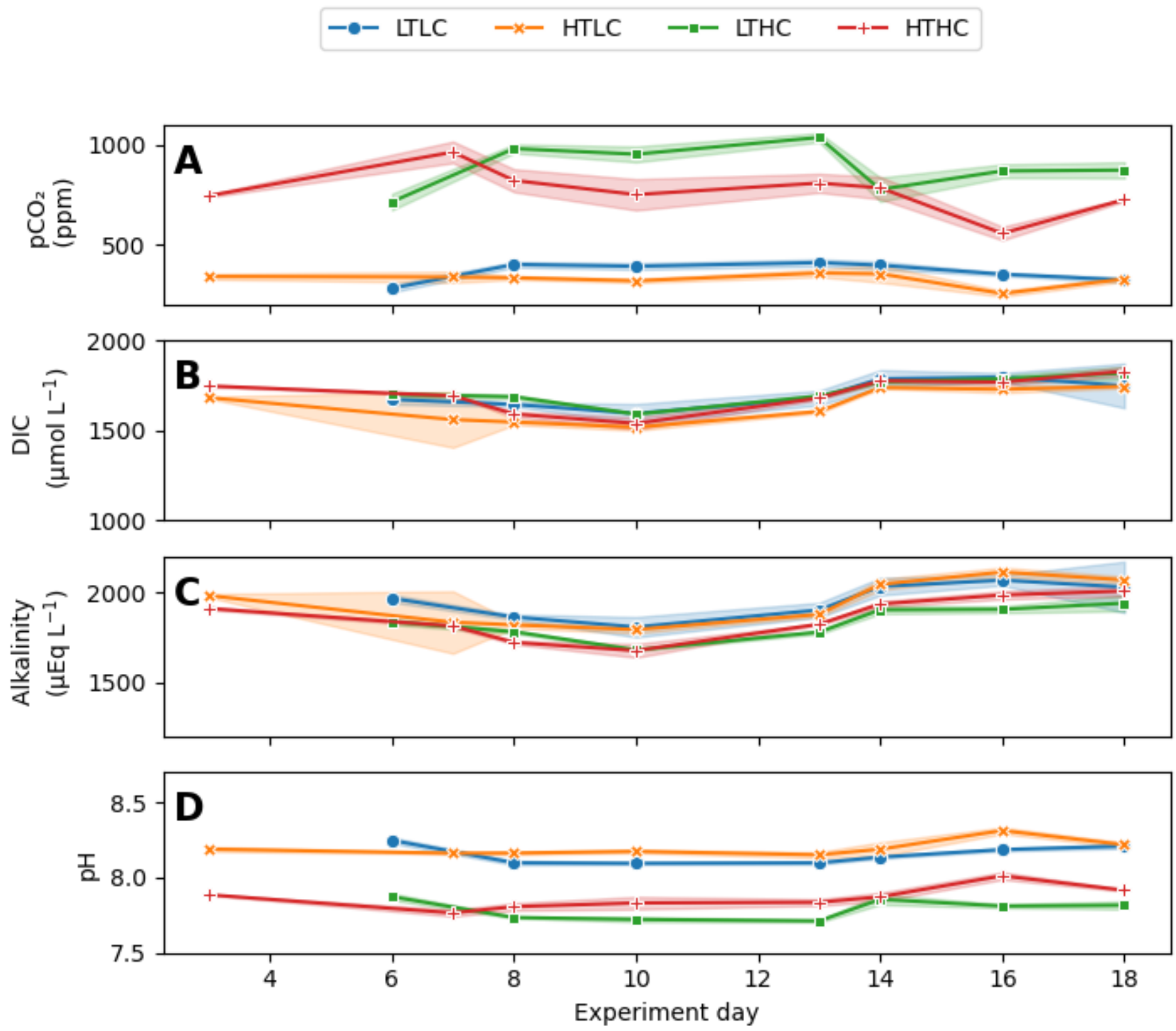

Figure S2: Carbon chemistry parameters over time, as measured before each transfer (some transfers are missing – due to faulty measurements of the elemental analyzer) for the different treatments. Shown are means with shades in the corresponding treatment colors representing standard deviation. (A) pCO_2 (calculated with PCO2SYS using pH and DIC), (B) Dissolved organic carbon - DIC (measured), (C) alkalinity (calculated with PCO2SYS using pH and DIC), and (D) pH (measured).

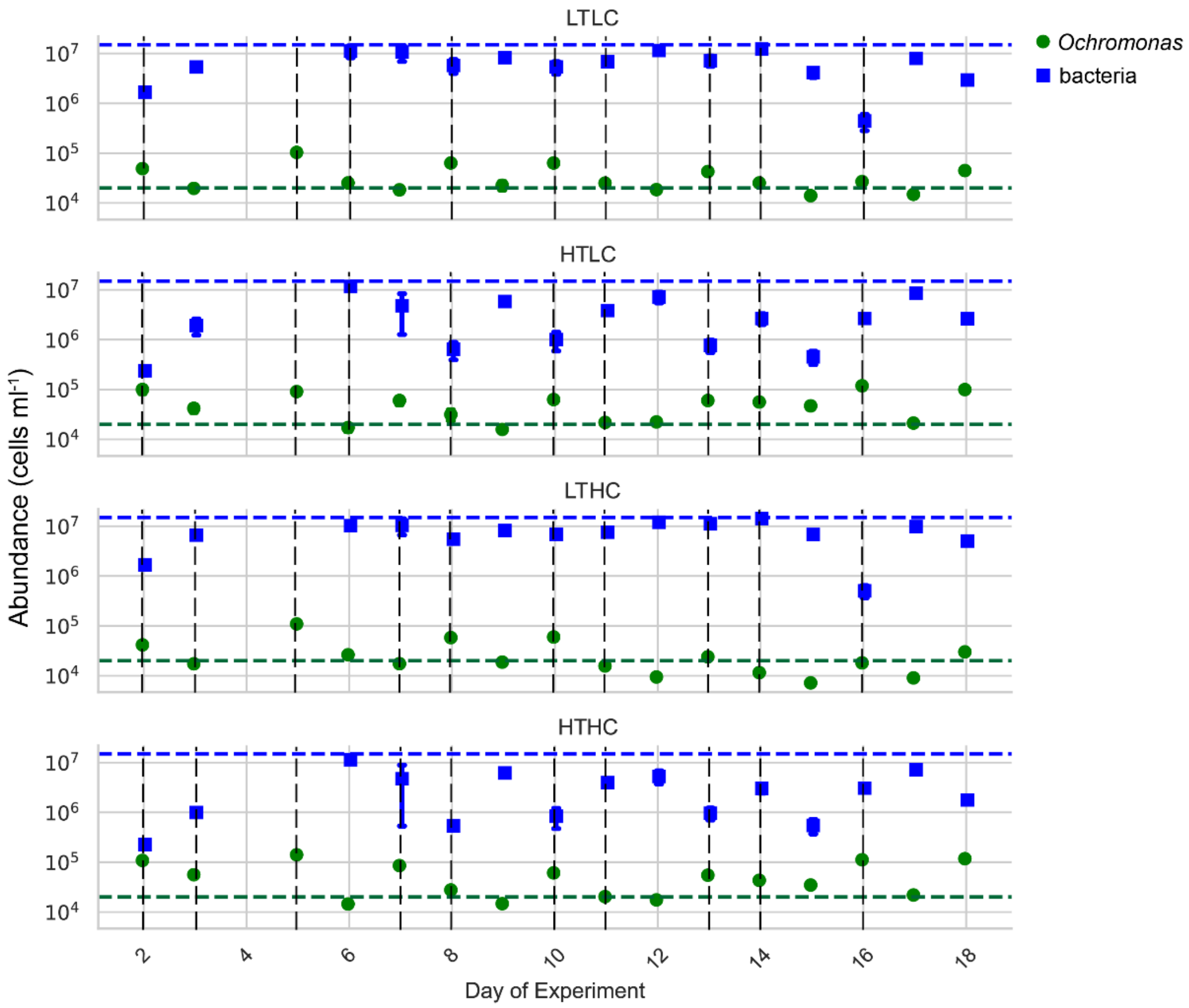

Figure S3: *Ochromonas* CCMP 2951 and bacterial abundances as measured live daily for the separate treatments. Measurements were conducted at the same hour every day, around two hours into the photic period. Vertical dashed lines indicate transfer day – in which the culture was diluted to a target 20,000 cells ml^{-1} (horizontal green dashed line) with freshly aerated media. Bacteria were counted each day. More bacteria were added to the cultures daily to a target concentration of $1.5*10^7$ cells mL^{-1} (horizontal blue dashed line). Error bars correspond to the standard deviation from the mean.

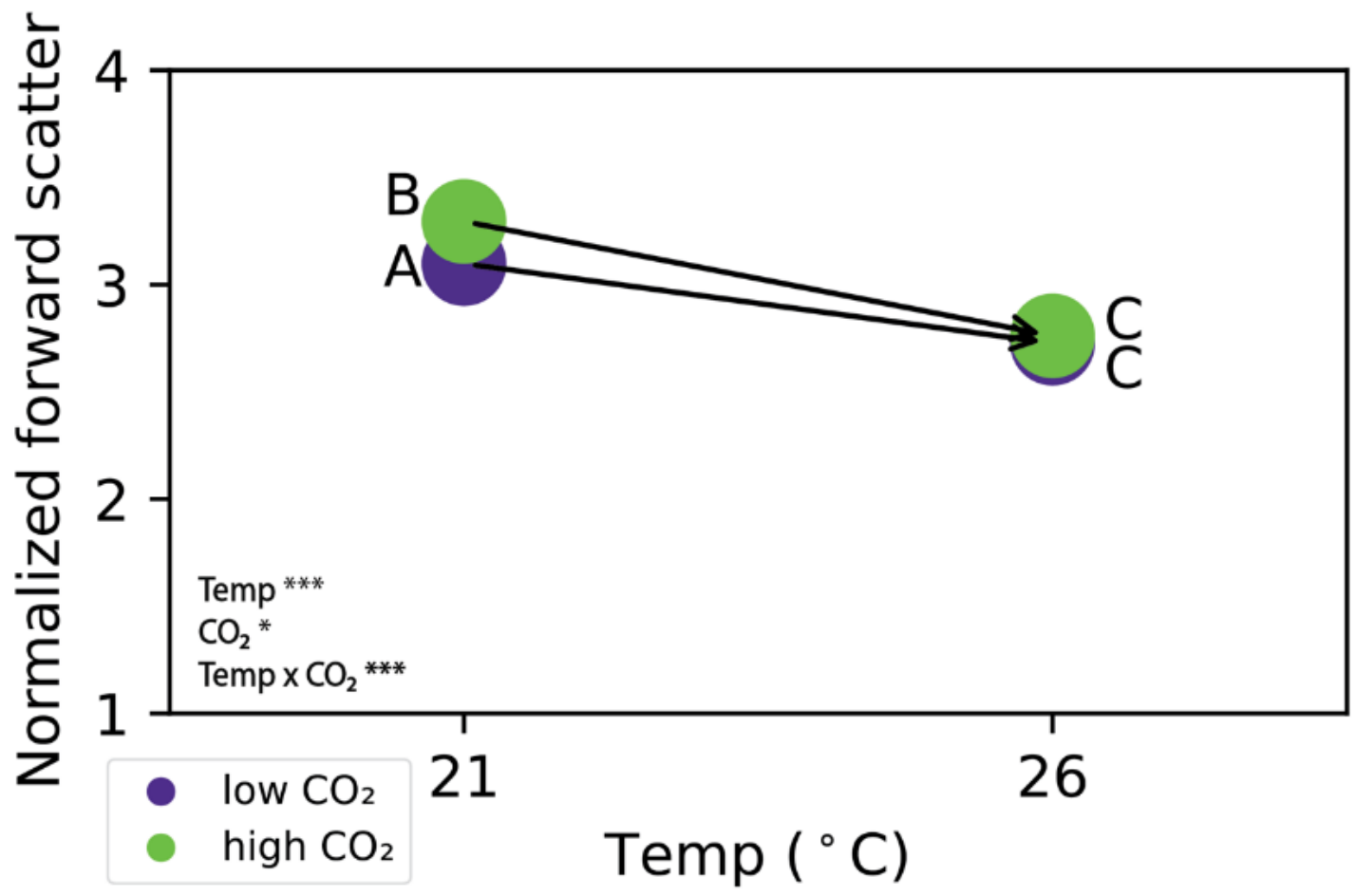

Figure S4: Mean forward scatter of *Ochromonas* CCMP2951 cells in the four experimental treatments normalized to beads forward scatter that were used as internal standards. The temperature of each treatment is indicated by the x-axis and the CO_2 level is indicated by colors. Arrows extend between the low and the high temperature of each CO_2. Error bars correspond to the standard deviation from the mean. The statistical significance of the two main effects (Temp and CO_2) and their interaction (Temp x CO_2) tested with Two-Way ANOVA are indicated (* = p<0.05, *** = p<0.001). Letters represent statistically significant differences between treatments based on Tukey's HSD pairwise post-hoc comparisons.

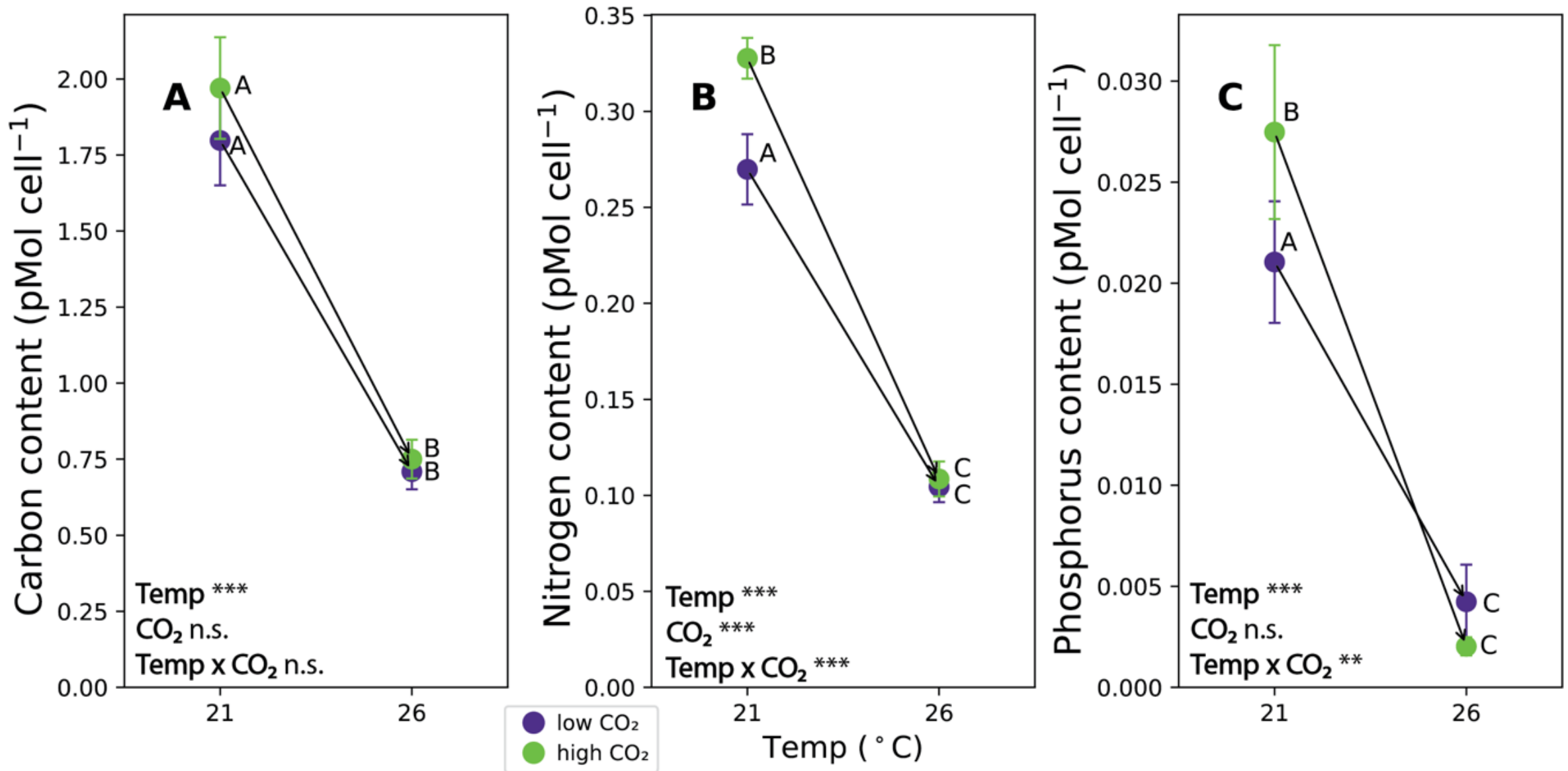

Figure S5: Elemental composition of *Ochromonas* CCMP2951: Cellular Carbon (A), Nitrogen (B) and Phosphorus content (C). The temperature of each treatment is indicated by the x-axis and the CO_2 level is indicated by colors. Arrows extend between the low and the high temperature of each CO_2. Error bars correspond to the standard deviation from the mean. The statistical significance of the two main effects (Temp and CO_2) and their interaction (Temp x CO_2) tested with Two-Way ANOVA are indicated (n.s. = $p>0.05$, ** = $p<0.01$, *** = $p<0.001$). Letters represent statistically significant differences between treatments based on Tukey's HSD pairwise post-hoc comparisons.

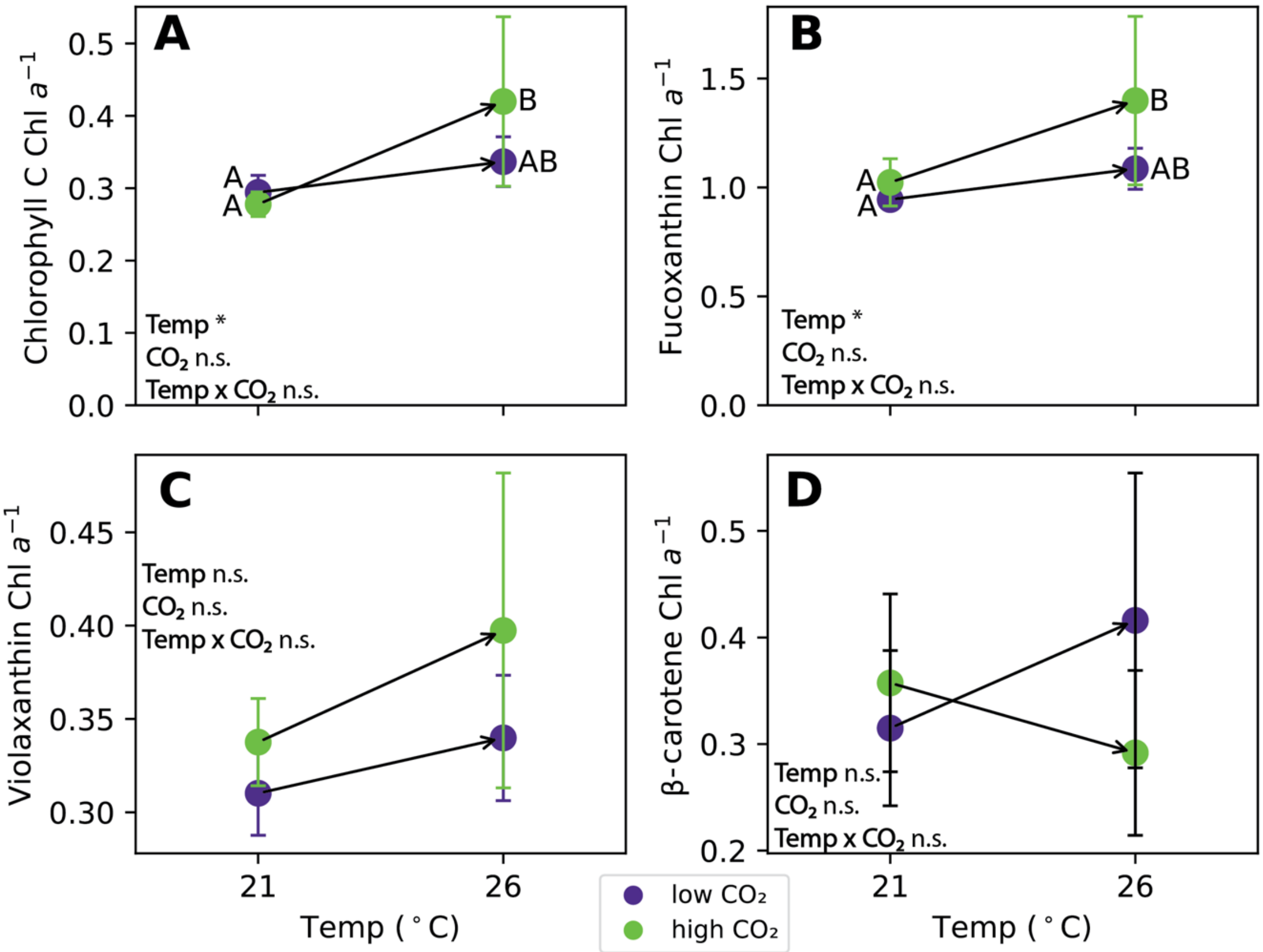

Figure S6: Relative pigment composition of *Ochromonas* CCMP2951 at the four experimental treatments: Chlorophyll *c* (*c*1+*c*2) (A), Fucoxanthin (B), Violaxanthin cycle pigments (Violaxanthin + Antheraxanthin + Zeaxanthin) (C) and β-carotene (D) expressed relative to Chlorophyll (Chl) *a* content. The temperature of each treatment is indicated by the x-axis and the CO_2 level is indicated by colors. Arrows extend between the low and the high temperature of each CO_2. Error bars correspond to the standard deviation from the mean. The statistical significance of the two main effects (Temp and CO_2) and their interaction (Temp x CO_2) tested with Two-Way ANOVA are indicated (n.s. = p>0.05, * = p<0.05). Letters represent statistically significant differences between treatments based on Tukey's HSD pairwise post-hoc comparisons.

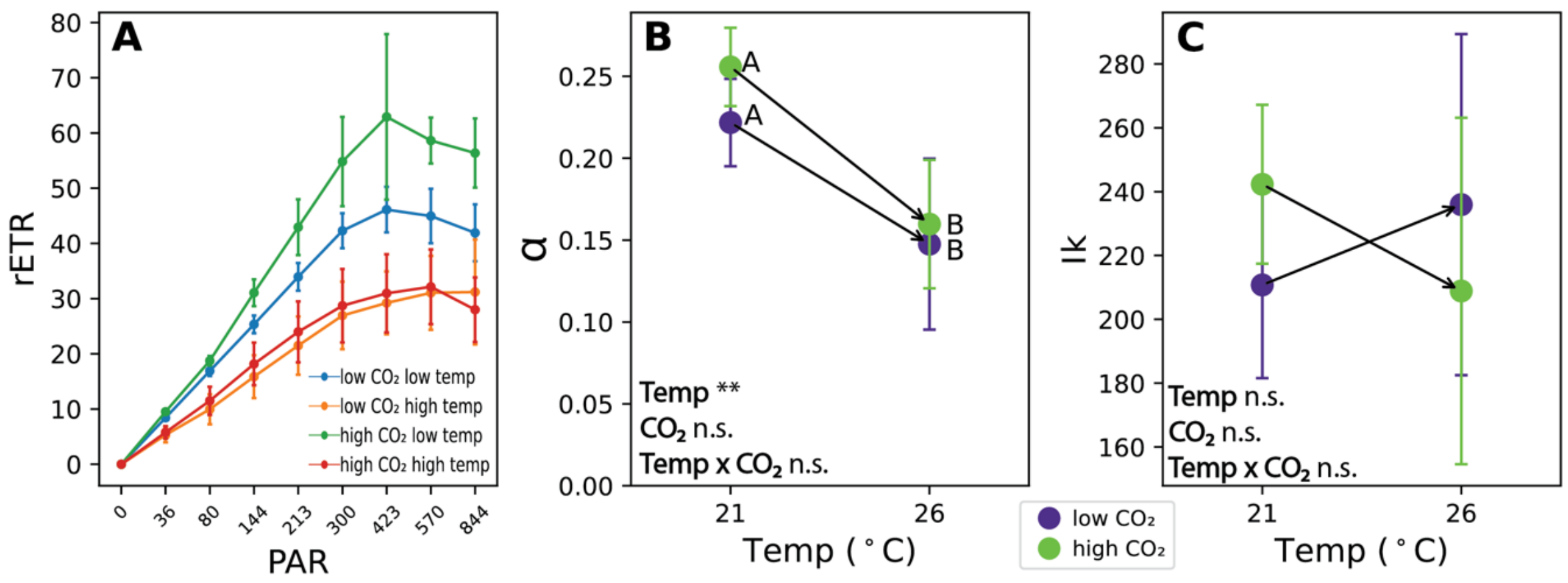

Figure S7: Rapid light curve results of *Ochromonas* CCMP2951 in the four experimental treatments. The relative electron transport rate (rETR) was calculated per light level (PAR) in the light curve for each experimental treatment (A). Alpha- the initial slope of the curve was extracted from a fitted model to each curve (B), as well as the half-saturation intensity, I_k (C). For panels B and C - the temperature of each treatment is indicated by the x-axis and the CO_2 level is indicated by colors. Arrows extend between the low and the high temperature of each CO_2. Error bars correspond to the standard deviation from the mean. The statistical significance of the two main effects (Temp and CO_2) and their interaction (Temp x CO_2) tested with Two-Way ANOVA are indicated (n.s. = $p>0.05$, ** = $p<0.01$). Letters represent statistically significant differences between treatments based on Tukey's HSD pairwise post-hoc comparisons.

Table S1: Results of three-way ANOVAs assessing the main effects of CO_2, temperature and the presence/absence of *Ochromonas* CCMP2951 and their interactions on the carbonate chemistry of experimental treatments. Statistically significant p-values are highlighted in bold font. DIC stands for dissolved inorganic carbon, temp for temperature.

Response variable	Term	$F_{(1,26)}$	P value
pCO_2	*Ochromonas*	8.071	**0.009**
pCO_2	CO_2	1356.997	**0.000**
pCO_2	temp	9.002	**0.006**
pCO_2	*Ochromonas* x CO_2	1.634	0.212
pCO_2	*Ochromonas* x temp	10.667	**0.003**
pCO_2	CO_2 x temp	1.593	0.218
pCO_2	*Ochromonas* x CO_2 x temp	4.091	0.054
pH	*Ochromonas*	12.360	**0.002**
pH	CO_2	874.772	**0.000**
pH	temp	7.032	**0.014**
pH	*Ochromonas* x CO_2	5.814	**0.023**
pH	*Ochromonas* x temp	6.429	**0.018**
pH	CO_2 x temp	0.461	0.503
pH	*Ochromonas* x CO_2 x temp	0.530	0.473
DIC	*Ochromonas*	0.590	0.450
DIC	CO_2	1.061	0.313
DIC	temp	1.845	0.186
DIC	*Ochromonas* x CO_2	0.001	0.977
DIC	*Ochromonas* x temp	0.125	0.727
DIC	CO_2 x temp	0.192	0.666
DIC	*Ochromonas* x CO_2 x temp	0.023	0.880
Alkalinity	*Ochromonas*	1.645	0.211
Alkalinity	CO_2	8.540	**0.007**
Alkalinity	temp	0.050	0.825
Alkalinity	*Ochromonas* x CO_2	0.259	0.615
Alkalinity	*Ochromonas* x temp	0.339	0.565
Alkalinity	CO_2 x temp	0.059	0.811
Alkalinity	*Ochromonas* x CO_2 x temp	0.028	0.868

Table S2: Results of two-way ANOVAs assessing the effects of CO_2, temperature and their interaction on different physiological parameters of *Ochromonas* CCMP2951. Statistically significant p-values are highlighted in bold font. Chl stands for Chlorophyll, rETR for relative Electron Transport rate, C for carbon, N for nitrogen, P for phosphorus, VAZ for violaxanthin + antheraxanthin + zeaxanthin and $C_{aut/het}$ for autotrophic divided by heterotrophic carbon acquisition balance.

Variable	CO_2		Temperature		Interaction term	
	$F_{(1,16)}$	p value	$F_{(1,16)}$	p value	$F_{(1,16)}$	p value
Growth rate (d^{-1})	6.290	**0.023**	16.128	**0.001**	0.946	0.345
mean cell volume (μm^3)	3.341	0.086	134.354	**<0.001**	2.495	0.134
Normalized forward scatter	31.164	**<0.001**	480.257	**<0.001**	15.550	**0.001**
Cellular C content (pg C $cell^{-1}$)	3.986	0.063	466.179	**<0.001**	1.507	0.237
Cellular N content (pg N $cell^{-1}$)	32.131	**<0.001**	1239.542	**<0.001**	24.318	**<0.001**
Cellular P content (pg P $cell^{-1}$)	2.866	0.110	287.208	**<0.001**	11.998	**0.003**
Molar C:N ratio	0.606	0.448	6.071	**0.025**	3.820	0.068
Molar C:P ratio	4.857	**0.043**	86.792	**<0.001**	12.167	**0.003**
Molar N:P ratio	6.700	**0.020**	78.537	**<0.001**	9.842	**0.006**
Carbon fixation (fg C μm^{-3} Och h^{-1})	7.439	**0.015**	0.315	0.582	2.075	0.169
Net photosynthesis (% gross)	0.4	0.53	107.913	**<0.001**	0.593	0.45
Chl *a* content (fg Chl μm^{-3} Och)	23.467	**<0.001**	4.980	**0.040**	0.682	0.421
Maximal rETR	3.609	0.077	55.131	**<0.001**	8.064	**0.012**

α (light curve slope)	1.924	0.184	26.095	**<0.001**	0.427	0.523
I_k (half-saturation intensity)	0.003	0.95	0.093	0.765	1.982	0.180
Chl *c* Chl a^{-1}	1.425	0.250	10.804	**0.004**	3.153	0.094
Fucoxanthin Chl a^{-1}	4.451	0.051	7.761	**0.013**	1.588	0.226
VAZ Chl a^{-1}	3.883	0.066	4.306	0.054	0.489	0.494
β-Carotene Chl a^{-1}	0.897	0.358	0.168	0.687	3.719	0.071
Grazing rate (fg μm^{-3} Och h^{-1})	7.035	**0.017**	45.746	**<0.001**	3.061	0.099
$C_{aut/het}$	35.594	**<0.001**	28.189	**<0.001**	17.031	**<0.001**

References

Eppley RW. 1971. Temperature and phytoplankton growth in the sea. *Fish. Bull.* **70:** 1063

Guillard RRL, Hargraves PE. 1993. *Stichochrysis immobilis* is a diatom, not a chrysophyte. *Phycologia* **32:** 234-236.

Norberg J. 2004. Biodiversity and ecosystem functioning: A complex adaptive systems approach. *Limnol. Oceanogr.* **49**: 1269-1277.

Padfield D, O'Sullivan HO, Pawar S. 2021. rTPC and nls.multstart: A new pipeline to fit thermal performance curves in R. *Methods Ecol. Evol.* **12:** 1138-1143.
